# Stability of Chloropyromorphite in Ryegrass Rhizosphere as Affected by Root-Secreted Low Molecular Weight Organic Acids

**DOI:** 10.1371/journal.pone.0160628

**Published:** 2016-08-05

**Authors:** Wei Wei, Yu Wang, Zheng Wang, Ruiming Han, Shiyin Li, Zhenggui Wei, Yong Zhang

**Affiliations:** 1 Jiangsu Provincial Key Laboratory of Materials Cycling and Pollution Control, School of the Environment, Nanjing Normal University, Nanjing, 210023, China; 2 Key Laboratory of Virtual Geographic Environment (Nanjing Normal University), Ministry of Education, Nanjing, 210023, China; 3 Jiangsu Center for Collaborative Innovation in Geographical Information Resource Development and Application, Nanjing, 210023, China; 4 School of Resources and Environment, Anqing Normal University, Anqing, 246011, China; 5 Department of Geographical Sciences, University of Alabama, Tuscaloosa, AL, 35487, United States of America; Friedrich Schiller University, GERMANY

## Abstract

Understanding the stability of chloropyromorphite (CPY) is of considerable benefit for improving risk assessment and remediation strategies in contaminated water and soil. The stability of CPY in the rhizosphere of phosphorus-deficient ryegrass was evaluated to elucidate the role of root-secreted low molecular weight organic acids (LMWOAs) on the dissolution of CPY. Results showed that CPY treatments significantly reduced the ryegrass biomass and rhizosphere pH. The presence of calcium nitrate extractable lead (Pb) and phosphorus (P) suggested that CPY in the rhizosphere could be bioavailable, because P and Pb uptake by ryegrass potentially provided a significant concentration gradient that would promote CPY dissolution. Pb accumulation and translocation in ryegrass was found to be significantly higher in P-sufficient conditions than in P-deficient conditions. CPY treatments significantly enhanced root exudation of LMWOAs irrigated with P-nutrient solution or P-free nutrient solution. Oxalic acid was the dominant species in root-secreted LMWOAs of ryegrass under P-free nutrient solution treatments, suggesting that root-secreted oxalic acid may be the driving force of root-induced dissolution of CPY. Hence, our work, provides clarifying hints on the role of LMWOAs in controlling the stability of CPY in the rhizosphere.

## 1. Introduction

The In situ immobilization of lead (Pb) in contaminated soils by phosphorus (P) -based materials including apatite minerals, nanosized hydroxyapatite, bone meal, bone char, inorganic phosphate, etc., has been considered as a promising remediation strategy, due to its low cost, high efficiency, easy-to-implement, and environmental friendly nature [1,2]. The addition of P to soils can induce rapid kinetic formation of lead phosphate precipitates, especially pyromorphites [Pb_5_(PO_4_)_3_(Cl, OH or F)], the most stable lead phosphate minerals encountered in nature under normal environmental conditions [3]. Thereby, other Pb species such as lead oxide, galena, anglesite, cerussite, and goethite adsorbed Pb, could react with various P-based materials and transform into pyromorphite through a dissolution-precipitation mechanism [4–7]. Within the pyromorphite mineral family, chloropyromorphite [CPY, Pb_5_(PO_4_)_3_Cl] is several orders of magnitude less soluble than the hydroxyl- and fluoro-pyromorphites [8]. Due to the ubiquity of chloride in soil, CPY is considered the dominant species of pyromorphite [9]. The formation of CPY has been confirmed under laboratory and in situ conditions through X-ray diffraction (XRD), scanning electron microscopy (SEM) coupled with energy dispersive X-ray (EDX), electron microprobe analysis (EMPA), and synchrotron radiation X-ray spectroscopy [10–15].

P-induced immobilization strategies for Pb are based on the low solubility of CPY. Therefore, studying the stability of CPY is of great importance for evaluating the remediation efficiency of Pb-contaminated soil by P. The geochemical stability of CPY has been extensively examined with pivotal research [8,16,17]. Nriagu [8] firstly suggested that the solubility product of CPY is approximately 10^−84^; however, a *K*_sp_ = 10^−25.05^ is more appropriate for soil pH in the range of 3 to 7 [18]. Nevertheless, CPY is several orders of magnitude less soluble than most common Pb minerals in soil, suggesting that the transformation of soil Pb to CPY would reduce the bioavailability and subsequent toxicity of Pb [16]. Until now, numerous studies have shown that CPY is a fairly stable Pb-mineral in aqueous solutions and bulk soils. For example, Scheckel and Ryan [19] studied the effects of aging and pH on the dissolution kinetics and stability of CPY using XRD, X-ray absorption fine structure (XAFS), and high resolution thermogravimetric analysis (HRTGA). Their results demonstrated that the thermostability of CPY increased with residence time, and that the dissolution rate of 1-day aged CPY was similar to the 1-year aged specimen, suggesting that the synthetic CPY could quickly achieve a low entropy state.

However, there is limited knowledge on the long-term stability of CPY in the rhizosphere where intense interactions with roots, soil organic matter, microbes, and root exudates including low molecular weight organic acids (LMWOAs), are expected [20]. LMWOAs concentrations in the rhizosphere are generally considered significantly higher than those in bulk soil solutions [21,22]. These LMWOAs not only play an important role in the activation of insoluble phosphates and theacquisition of phosphorus [21,23–25], but also participate in the uptake, translocation and accumulation of heavy metals by plants [26–28]. Root-induced phosphate dissolution in the rhizosphere of white lupins has been reported by Hinsinger and Gilkes [29]. In addition, Calvaruso et al. [30] found that the dissolution of apatite and the release of calcium, phosphorus, zinc and rare earth elements could be facilitated by LMWOAs originating from tree roots and root-associated microorganisms. Moreover, metal-tolerant fungi could dissolve toxic metal-phosphate minerals such as hopeite and pyromorphite, and thus enhance the mobility of zinc and lead by acidolysis and complexolysis through the secretion of LMWOAs [31,32]. Furthermore, in our previous work [26], LMWOAs were found to promote the uptake and transportation of heavy metals. On the other hand, it is widely accepted that P deficiency and Pb stress could induce the secretion of LMWOAs in certain plant species [33–36]. Recently, Abbaspour et al. [37] and Abbaspour and Arocena [38] reported that vegetation could potentially release Pb from pyromorphite into the environment, and CPY could be transformed into lanarkite on the root surface of Indian mustard, suggesting that rhizosphere processes could promote the CPY dissolution. However, they did not collect root exudates or analyze LMWOAs in the rhizosphere; that is, a direct observation is still missing on the relationship between CPY dissolution and LMWOAs exudation in the rhizosphere. Therefore, the stability of CPY in the rhizosphere, especially the role of rhizosphere LMWOAs in the dissolution of CPY and uptake of Pb and P by plants remain obscure motivating further examination by this study.

The objective of this work is to investigate the phytoavailability of CPY to ryegrass grown in sand culture and evaluate the roles of root-secreted LMWOAs on the stability of CPY in the rhizosphere.

## 2. Materials and Methods

### 2.1 Preparation of chloropyromorphite

**Fig 1:**
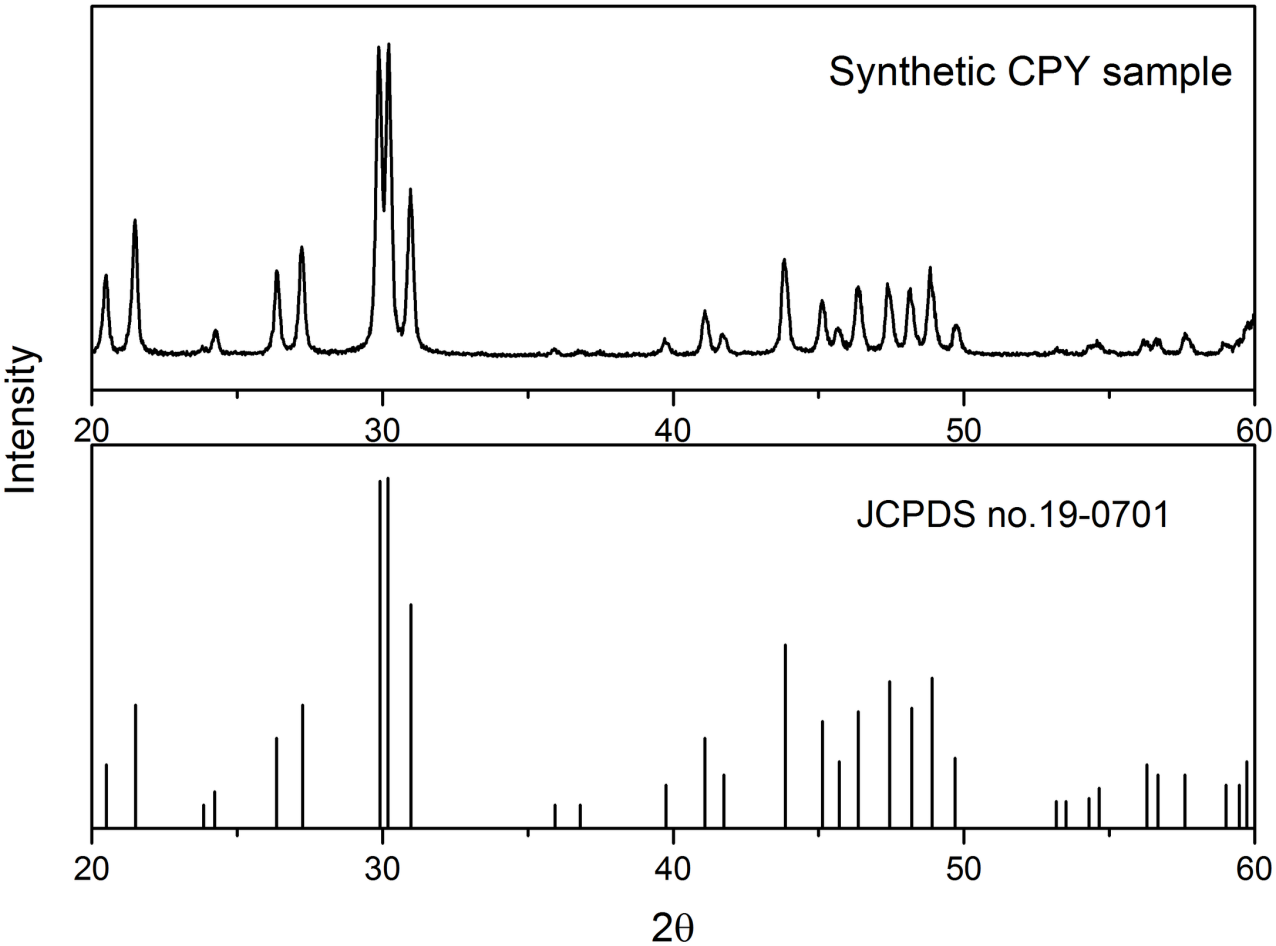
The XRD patterns of the synthetic CPY and the reference pattern of pure CPY (JCPDS no. 19–0701).

The CPY used in this study was synthesized in our laboratory according to the method described by Sayer et al. [31] with modification. In brief, Pb(NO_3_)_2_ (200 mL of 0.5 mol L^-1^) was mixed with 200 mL of a solution containing 0.3 mol L^-1^ Na_2_HPO_4_ and 0.1 mol L^-1^ NaCl at ambient temperature. When cooled, the precipitate was isolated by filtration, washed in deionized water and dried at 60°C. Identification of CPY was confirmed by chemical analysis and X-ray diffraction (Fig 1).

### 2.2 Plant cultivation and experimental design

The quartz sand ranged from 0.45 to 1 mm in grain diameter and was initially washed with tap water, then treated with 2 mol L^-1^ HCl for 24 h. It was again washed with tap water until the water was neutral and then sterilized at 170°C for 2 h in an oven. Pots for growing the ryegrass were each filled with 500 g of the sand.

Ryegrass (*Lolium perenne* L.) was chosen because of its tolerance and ability to accumulate Pb [39], as well as its ability to secret LMWOAs under P deficiency or heavy metal stress [40]. Ryegrass seeds were obtained from Jiangsu Academy of Agricultural Sciences, China. To inhibit microbe growth, the seeds were surface-disinfected by soaking them in a 3% (v/v) solution of hydrogen peroxide for 15 min and rinsed with sterile distilled water. They were germinated on pledgets in an illuminated incubator at 25°C. The seedlings were transplanted to greenhouse pots (4 plants per pot) filled with either control or CPY spiked quartz sand after 2 days of emergence, making the beginning of cultivation. The greenhouse was maintained at 20 to 25°C during the day and 10 to 15°C at night. Relative humidity was controlled at 60%.

The pots were divided into two groups (Table 1) and watered daily with 25 mL 1/2 strength modified Hoagland nutrient solution. One group (spiked with 0, 1.31 and 3.93 g CPY) was watered with a P-nutrient solution, while the other group (spiked with 1.31 and 3.93 g CPY) was watered with a P-free nutrient solution in which KCl was used in place of KH_2_PO_4_ for the nutrient solution as recommended by Abbaspour et al. [37]. Each treatment was performed in four replications. The modified Hoagland nutrient solution contained the following nutrients: 1 mmol L^-1^ KNO_3_, 1.5 mmol L^-1^ Ca(NO_3_)_2_, 0.5 mmol L^-1^ MgSO_4_, 20 μmmol L^-1^ KH_2_PO_4_, 1 μmol L^-1^ H_3_BO_3_, 0.7 μmol L^-1^ MnSO_4_, 0.5 μmol L^-1^ ZnSO_4_, 5 μmol L^-1^ Fe(II)-EDTA,0.01 μmol L^-1^ (NH_4_)_6_Mo_7_O_24_, and 0.075 μmol L^-1^ CuSO_4_. Moisture in each pot was kept to 60–70% water holding capacity throughout the experiment. The plants were collected after a 30-day cultivation.

**Table 1:** The rates of Pb and P addition to the treatments.

Treatments*	Nutriment solution	CPY added	Pb contained	P contained
		g/pot		
P0PC1PC3PWC3NC1NC3NWC3	PP-free	01.313.933.931.313.933.93	0133133	00.110.270.270.110.270.27

* P: pots irrigated with the P-nutrient solution; N: pots irrigated with the P-free nutrient solution; W: pots without the plant; C: pots containing CPY; 1 or 3 g Pb as CPY in each pot.

### 2.3 Collection of root exudates

The resulting plant roots were washed in deionized water and then submerged in a 100 mL solution containing 1 mmol L^-1^ of CaSO_4_ and 0.5 mg L^-1^ of thymol, a nontoxic concentration for the plant [25]. After 4-h growth, root exudates were collected, evaporated until dry under reduced pressure at 45°C, dissolved in 5 mL of distilled water, and then stored in a refrigerator at -20°C before undergoing reversed-phase high performance liquid chromatography (RP-HPLC) analysis.

### 2.4 Biomass determination

After harvest, each plant was washed with tap water and then divided into root and shoot fractions, placed in paper bags and dried in an air circulation oven at 80°C to a constant weight in order to determine biomass production.

### 2.5 pH measurements

The sand culture of the pots was separated into rhizosphere and bulk. Sand that remained adhered to the roots after gentle shaking was sampled as rhizosphere sands by operating definition [41]. The pH of the samples was determined in 1:2.5 sand-water suspension using a pH meter.

### 2.6 Extraction of available Pb and P

Calcium nitrate (2 g of sand sample and 10 mL of 0.1 mol L^-1^ Ca(NO_3_)_2_) extraction was used to determine the bioavailability of Pb and P in the sand samples, because weak-electrolyte extraction using calcium nitrate has shown potential as a surrogate measure of bioavailability of cadmium, zinc, and Pb in soil [42].

### 2.7 Pb and P uptake by ryegrass

Vegetal dry samples were mill ground and digested with a mixture of HNO_3_ and HClO_4_ (87:13 v/v). Pb and P concentrations in the digested solution were measured using inductively coupled plasma atomic emission spectroscopy (ICP-AES, Optimal 2100DV, Pekin Elmer).

### 2.8 LMWOAs determination

LMWOAs in the root exudates were determined by RP-HPLC after solid-phase extraction with nanosized hydroxyapatite as described previously [26,43]. The HPLC analyses were carried out on an Agilent 1100 liquid chromatograph equipped with a UV-Vis detector (Agilent, USA). A Thermo Syncronis aQ C18 column (150 × 4.6 mm, 5 μm particle size) was used. The mobile phase was a buffer solution containing 50 mmol L^-1^ (NH_4_)_2_HPO_4_ adjusted to a pH of 2.5 with H_3_PO_4_; it was filtered through a 0.45 μm membrane filter supplied by XinYa Corporation (Shanghai, China). This mobile phase solution must be prepared fresh daily. Separation was carried out by isocratic elution with a flow rate of 1.0 mL min^-1^ while the column temperature was maintained at a constant 25°C. The optimum wavelength for determination was 214 nm with a sensitivity of 0.02 absorbance unit, full scale. The injection volume was 20 μL and each sample was injected in triplicate. The determination of acids was done in peak area mode.

### 2.9 Statistical analysis

Data was expressed as means ± standard deviation (SD). All statistical analyses were performed with SPSS 16.0 for Windows. One-way ANOVA was used to determine any significant differences among the treatments. Differences between individual means were tested using the least significant difference test at the 0.05 significance level.

## 3. Results

### 3.1 Biomass production of ryegrass

**Fig 2:**
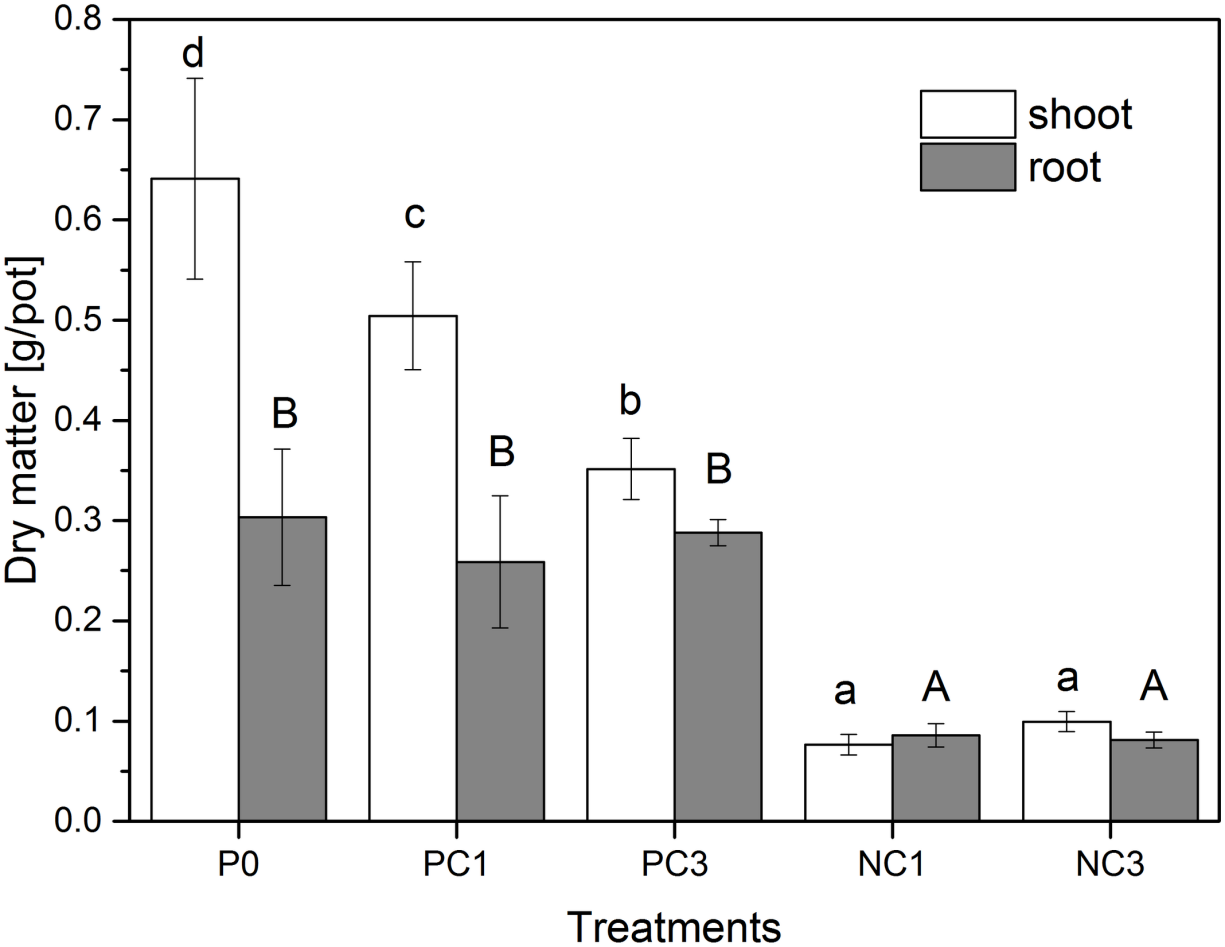
Effect of CPY treatments on the dry weight of ryegrass. Error bars represent standard deviation.

Fig 2 shows biomass production of ryegrass (dry weight) treated with P-nutrient solution and P-free nutrient solution spiked with different CPY levels. It indicated that CPY treated seedlings showed lower values of both shoot and root dry weights than the control (P0). In comparison to the control, shoot and root dry weights decreased by 21.3% and 14.6% for PC1, and 45.1% and 5.0% for PC3, respectively. Furthermore, in pots treated with P-free nutrient solution, the addition of CPY (NC1 and NC3) significantly decreased the shoot and root dry weights of ryegrass. Shoot and root dry weight decreased by 88.1% and 71.7% for NC1, and 84.5% and 73.3% for NC3, as compared with that of the control.

### 3.2 pH values in the bulk and rhizosphere of ryegrass

**Fig 3:**
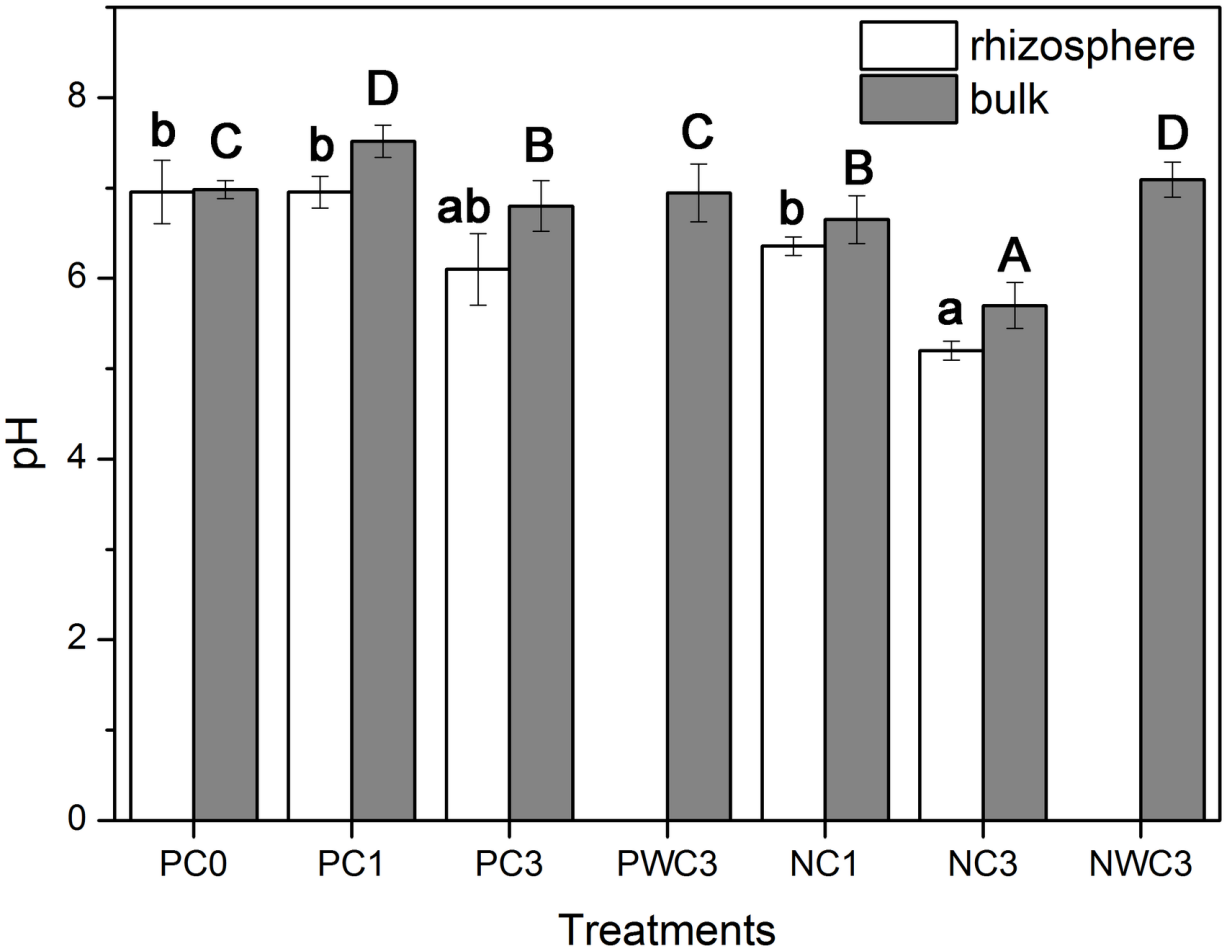
Effect of CPY treatments on pH values in the bulk and rhizosphere of ryegrass. Error bars represent the standard deviation.

Values for pH in the rhizosphere and bulk ryegrass under different treatments are shown in Fig 3. Results indicated that ryegrass vegetation could significantly affect rhizosphere and bulk pH when compared with non-vegetated treatments (PWC3 and NWC3). Roots increased bulk pH by 0.2–0.7 and 0.3–0.5 units in pots treated with P-nutrient solution and P-free nutrient solution, respectively. Fig 3 also showed that the ryegrass rhizosphere pH was slightly lower than bulk pH under CPY treatments.

The results of this study also show that the ryegrass rhizosphere pH was slightly lower than bulk pH under CPY treatments, which is in contrast to the results reported by Abbaspour and Arocena [38] even though more NO_3_^-^ was supplied than NH_4_^+^. Similarly, Grinsted et al. [44] had found that rhizosphere pH could decrease even though NO_3_^-^ was supplied exclusively, suggesting that other processes such as organic anion release, root exudation and respiration, and redox-coupled processes might be involved in root-induced pH changes in the rhizosphere [45]. There is considerable evidence that P deficiency or Pb stress could induce the secretion of LMWOAs by some plants [33–36]. For example, Qiao et al. [40] indicated that ryegrass was capable of releasing large amounts of organic acids into the rhizosphere in response to Pb stress. Therefore, root-secreted LMWOAs might play a more significant role than other factors, in decreasing ryegrass rhizosphere pH, due to P deficiency and Pb stress under CPY treatments.

### 3.3 Calcium nitrate-extractable P and Pb

**Fig 4:**
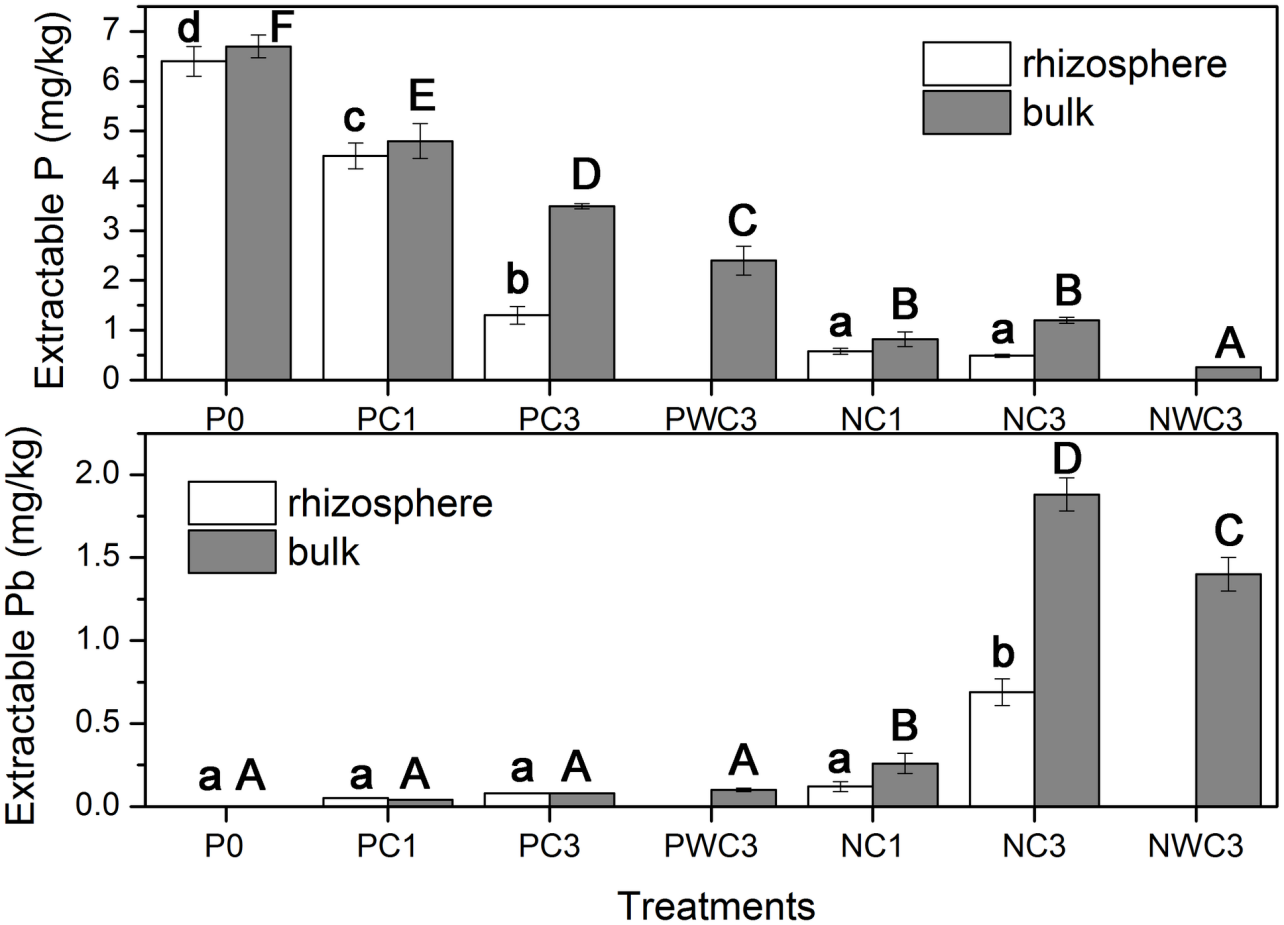
Calcium nitrate-extractable P and Pb in the bulk and rhizosphere of ryegrass. Error bars represent the standard deviation.

Calcium nitrate-extractable P and Pb in the bulk and rhizosphere of ryegrass are shown in Fig 4. It was clear that extractable P in pots treated with P-nutrient solution were much higher than in pots treated with P-free nutrient solution. In addition, the content of extractable P in pots treated with P-nutrient solution was the lowest in PWC3, while that in P-free treatments was the lowest in NWC3.

Fig 4 also shows that Ca(NO_3_)_2_ extractable Pb was less than 0.1 mg kg^-1^ in pots treated with P-nutrient solution, which is much lower than those treated with P-free nutrient solution. However, in pots treated with P-free nutrient solution, Ca(NO_3_)_2_ extractable Pb levels were 0.15, 0.26 mg kg^-1^ (NC1) and 0.69, 1.88 mg kg^-1^ (NC3) for the rhizosphere and bulk matter, respectively. On the other hand, there was a significant difference in the concentration of Ca(NO_3_)_2_ extractable Pb of unplanted pots between PWC3 and NWC3. A higher concentration of Ca(NO_3_)_2_ extractable Pb was found in NWC3 (1.40 mg kg^-1^) than PWC3 (0.09 mg kg^-1^).

### 3.4 Uptake of Pb and P by ryegrass

**Fig 5:**
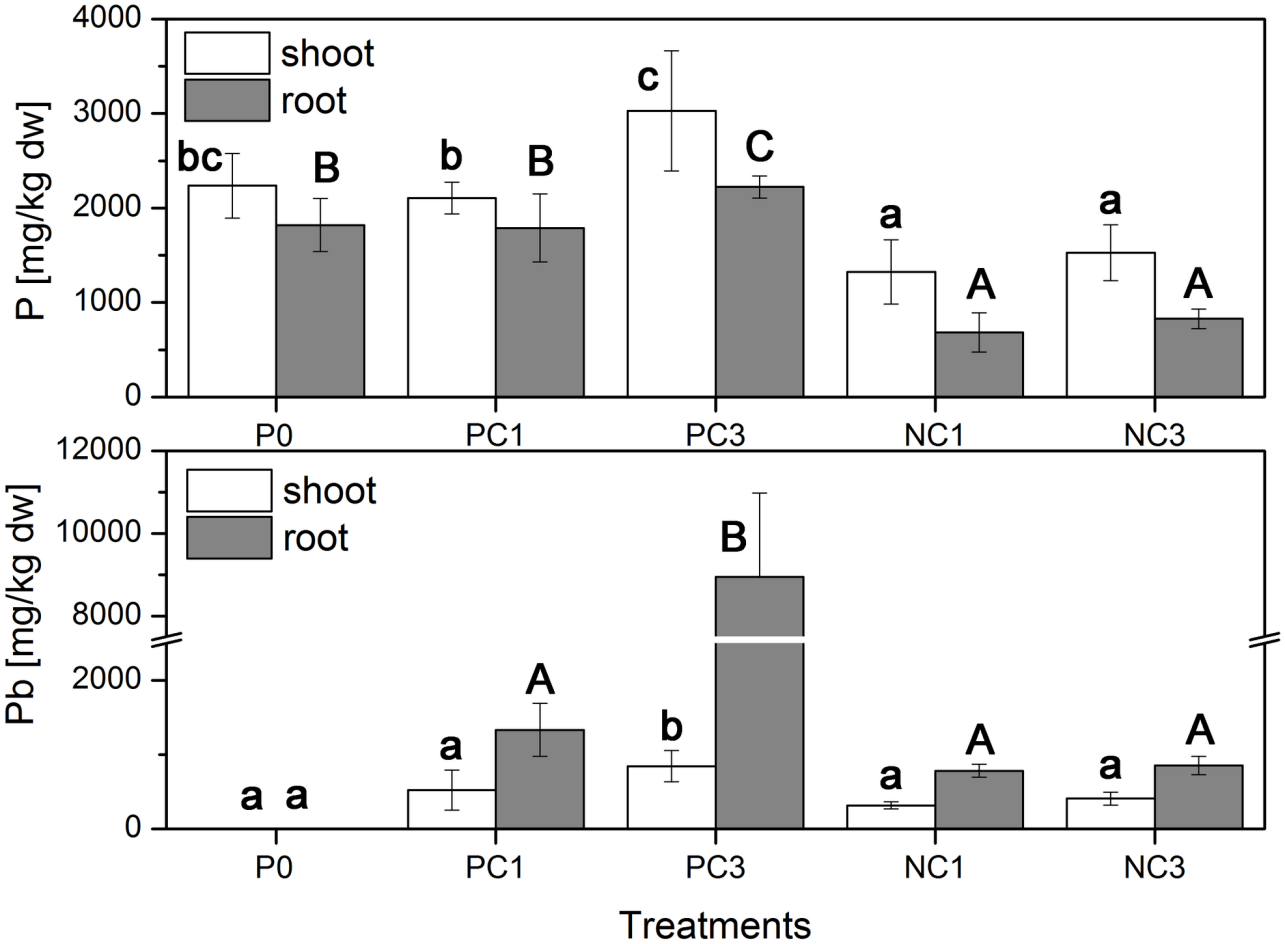
Effect of CPY treatments on the accumulation of P and Pb in the shoot and roots of ryegrass. Error bars represent the standard deviation.

The uptake of Pb and P by ryegrass under CPY treatments is shown in Fig 5. On one hand, compared with the control (P0), the shoot P content in NC1 and NC3 significantly decreased by 40.8% and 31.7%, respectively. On the other hand, compared with P0, the root P content in NC1 and NC3 significantly decreased by 62.4% and 54.5%, respectively. In addition, there was no significant difference in shoot and root P content between control (P0) and PC1; however, shoot and root P content increased by 26.1% and 18.2%, respectively, in PC3 treatment as compared with P0.

The uptake of Pb by ryegrass spiked with sparingly soluble CPY is shown in Fig 5. It was clear that the shoot Pb content in all CPY treatments was significantly lower than corresponding root Pb content. The results in Fig 4 also show that the uptake and accumulation of Pb in ryegrass tissue vary depending on the P source added (Table 1). Pb uptake in shoot and roots was 523.8 and 1334.1 mg kg^-1^ for PC1, and 845.3 and 8954 mg kg^-1^ for PC3, respectively. In contrast, Pb uptake in shoot and roots for NC1 was 39.3% and 41.2% lower than PC1, respectively. While the accumulation of Pb in shoot and roots for NC3 was 51.5% and 90.5% lower than PC3, respectively. Moreover, there was no significant difference in the accumulation of Pb between NC1 and NC3, which were irrigated with P-free nutrient solution.

### 3.5 Root secretion of LMWOAs by ryegrass

**Fig 6:**
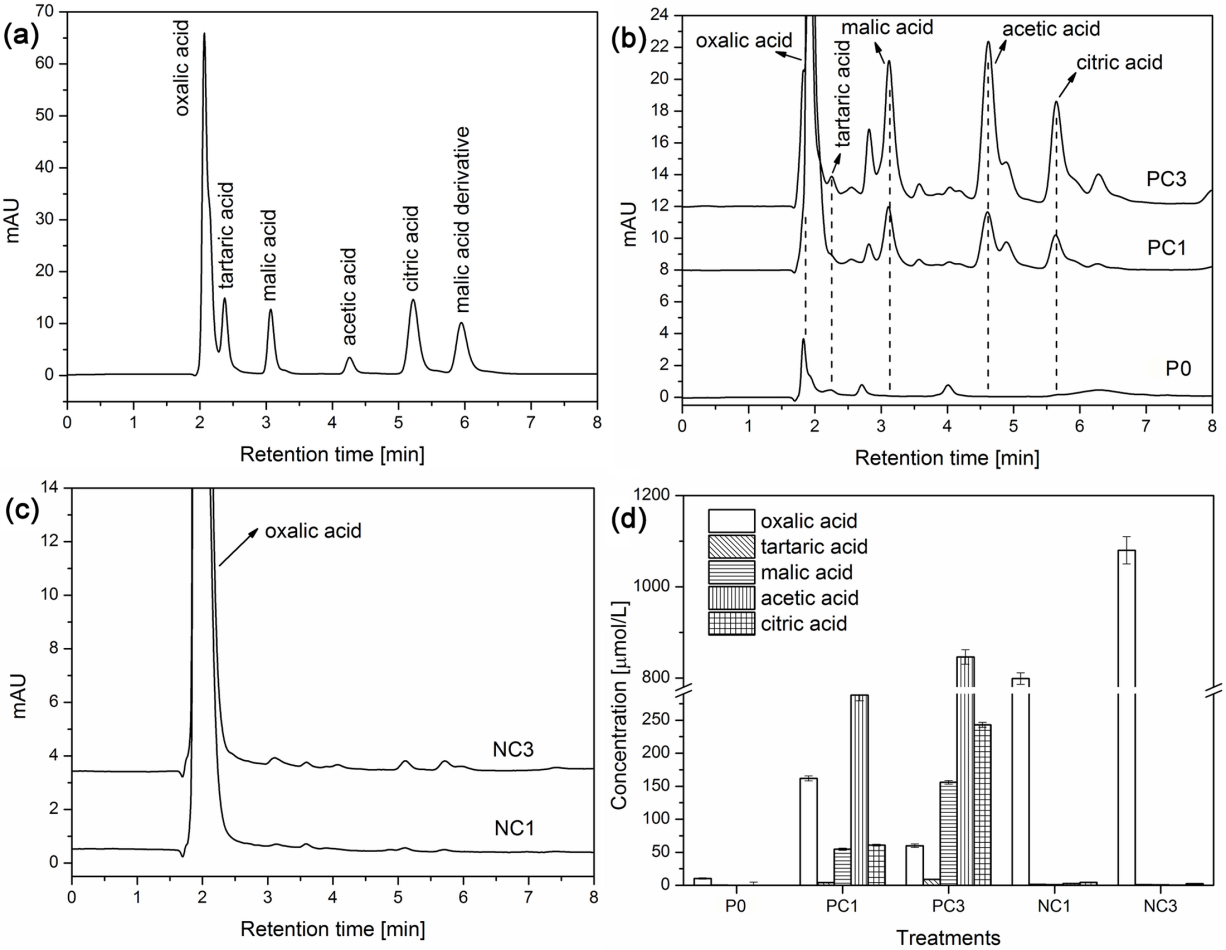
(a) RP-HPLC chromatogram of a standard mixture of five organic acids. Effect of CPY treatments on root-secreted LMWOAs by ryegrass treated with (b) P-nutrient solution and (c) P-free nutrient solution. (d) Concentrations of root-secreted LMWOAs by ryegrass irrigated with P-nutrient solution and P-free nutrient solution under CPY treatments.

Fig 6a shows the RP-HPLC chromatogram of a mixed standard solution of five organic acids (oxalic, tartaric, malic, acetic, and citric acids). It was evident that these organic acids could be efficiently separated under present HPLC conditions. The HPLC chromatogram of ryegrass root exudates under CPY treatments irrigated with P-nutrient solution and P-free nutrient solution are shown in Fig 6b and 6c, respectively. These results indicated that oxalic, malic, acetic, and citric acids in root exudates could be separated and identified in one injection by HPLC. Tartaric acid could not be detected due to its low concentration.

As shown in Fig 6b, concentrations of root-secreted organic acids in PC1 and PC3 increased significantly compared with the control P0. However, in pots treated with P-free nutrient solution, concentrations of root-secreted oxalic acid in NC1 and NC3 reached 798.7 and 1080 μmol L^-1^, respectively (Fig 6c and 6d), which were much higher than other organic acids.

## 4. Discussion

### 4.1 Stability of CPY in ryegrass rhizosphere

To achieve the remediation of a Pb-contaminated environment, the transformation of Pb to CPY with extremely high stability, using phosphorus-bearing materials, has gained extensive attention and recognition over the world [18]. However, although CPY was considered to be fairly stable in bulk soil, the long term stability of CPY in rhizosphere was not fully understood.

A decrease in the dry weight of ryegrass (Fig 2) induced by CPY treatments was in agreement with Abbaspour et al. [37] who reported that the addition of CPY significantly decreased the dry weight production of *Brassica juncea* and *Medicago sativa* treated with P-free nutrient solution. In the present study CPY decreased the dry weight of plants irrigated with P-containing nutrient solution in comparison with controls. Although there was no CPY-free and P-free controls, the dry weight of plants irrigated with P-free nutrient solution were lower than plants irrigated with P-containing nutrient solution. In most of the previous studies, a decrease in dry plant weight was reported under Pb treatment [46,47]. The results also indicated that although CPY was considered sparingly soluble and a less toxic Pb-bearing mineral [8,18,19], it still exhibited some phytotoxicity towards ryegrass just like free Pb ions, as reflected by the reduced biomass. However, it was clear that P deficiency or lower P bioavailability reduced growth more than Pb uptake.

Changes in bulk and rhizosphere pH values (Fig 3) were ascribed to an imbalance between soluble cations and anions in the rhizosphere because soil pH was mainly controlled by the presence of soluble or exchangeable cations and anions [48]. There was considerable evidence that P deficiency or Pb stress could induce the secretion of LMWOAs by some plants [33–36]. For example, Qiao et al. [40] indicated that ryegrass was capable of releasing large amounts of organic acids into the rhizosphere in response to Pb stress. Therefore, root-secreted LMWOAs might play an important role in decreasing rhizosphere pH, which further affected the stability of CPY in the ryegrass rhizosphere.

The higher values of Ca(NO_3_)_2_ extractable P in vegetation treatments suggested that rhizosphere processes could promote the dissolution of CPY. Similarly, Hinsinger and Gilkes [29] have reported root-induced dissolution of phosphate rock in the rhizosphere of white lupins. Furthermore, in all the vegetation treatments, Ca(NO_3_)_2_ extractable P levels in the rhizosphere were lower than those in the bulk matter, which might be due to the acquisition of P by ryegrass. On the other hand, considerable amounts of Ca(NO_3_)_2_ extractable P were found in pots treated with P-free nutrient solution, suggesting that CPY might not be as stable as expected by its *K*_sp_, and could be partly dissolved in rhizosphere processes. Similar results were also found by Sayer et al [31], who reported that CPY could be solubilized by organic acid producing fungi and transformed into lead oxalate dihydrate. The Ca(NO_3_)_2_ extractable Pb in pots treated with P-free nutrient solution is considered to have originated from the Pb adsorbed on the CPY surface. As reported by Martínez et al. [49], there were excessive Pb ions on the CPY surface due to oversaturation with respect to CPY synthesis, and thus the Ca(NO_3_)_2_ extractable Pb in pots treated with P-free nutrient solution was higher than in those treated with P-nutrient solution. In contrast, less Ca(NO_3_)_2_ extractable Pb in pots irrigated with P-nutrient solution were attributed to the probable precipitation of CPY by excessive P.

Compared with P0, the relatively low P content in the shoots and roots of ryegrass treated with P-free nutriment solution indicated that the P supply had poor efficiency and that CPY had high stability. However, considerable amounts of P in shoot and roots (NC1 and NC3) strongly suggest that, in the absence of other soluble sources of phosphate, plants could induce the dissolution of CPY while P remained free and available for plant uptake. In contrast, the irrigation of P-nutrient solution could improve P uptake by ryegrass under CPY treatments.

The accumulation of Pb by ryegrass has been demonstrated in numerous reports [50,51]. However, the uptake and translocation of Pb by plants could be simultaneously affected by various factors, such as plant species, growth conditions and Pb speciation. Lower contents of shoot Pb in the CPY treatments were consistent with the previous study where Pb was preferred to accumulate in the roots with little translocation to the shoots [52]. Previous research suggested that some plants used an exclusion mechanism to accumulate Pb in their roots and limit its transport to the shoots. Electron microscopy and XRD studies indicated that Pb accumulated in the root by binding to root cell walls or by precipitating as lead phosphate [13]. Uptake and accumulation of Pb in the ryegrass tissues varied depending on the P source used. It was worth noting that although the Ca(NO_3_)_2_ extractable Pb (Fig 4) in NC1 and NC3 were higher than PC1 and PC3, respectively, the shoot and root Pb contents (Fig 5) were lower in NC1 and NC3 than that in CPY treatments irrigated with P nutriment solution, which suggested that Pb accumulation and translocation in the ryegrass was significantly higher in P-sufficient conditions than in P-deficient conditions. This may have occurred due to the large, most supreme under P-sufficient conditions. Similar findings were reported by Abbaspour et al. [37], who also founded that P supplied by hydroxyapatite or P-nutrient solution was more effective in Pb acquisition by Indian mustard and alfalfa from CPY than P deficient conditions. Furthermore, lower values of Ca(NO_3_)_2_ extractable Pb in the growing medium and higher Pb accumulation by ryegrass in PC1 and PC3 might also be attributable to the uptake, translocation and accumulation of Pb by ryegrass, which has been widely reported for its tolerance and ability to accumulate Pb [39,50,51].

### 4.2 Effect of CPY on root secretion of LMWOAs by ryegrass

LMWOAs are natural products of root exudates, microbial secretions, and plant and animal residue decomposition in soils [20]. It is well known that the secretion of LMWOAs (such as oxalic, tartaric, malic, acetic and citric acids) by roots plays a significant role in root nutrient acquisition, mineral weathering, microbial chemotaxis and metal detoxification [53]. Therefore, analyses of root-secreted LMWOAs by Ryegrass under CPY treatments are essential to elucidate the role of LMWOAs in the dissolution of CPY and to evaluate the stability of CPY in the rhizosphere.

Previous studies have well documented that root exudation of LMWOAs, mainly including acetic, citric, fumaric, malic, oxalic, and succinic acids, were enhanced in many plant species under P deficiency or metal stress [36,40,53]. However, considering the irrigation of P-nutrient solution in these treatments, the enhanced root exudation of LMWOAs in PC1 and PC3 was probably due to the Pb-containing CPY stress, suggesting the potential phytotoxicity of CPY. There exists overwhelming evidence implying that some plants could directly modify the rhizosphere via root exudation of LMWOAs in order to gain access to previously unavailable P reserves [21,23]. For instance, P-deficient white lupin and rape roots exuded striking amounts of citric and malic acid, which could be a highly effective strategy for plant roots to enhance P uptake from insoluble phosphate [23,54]. The root-secreted organic acids resulted in the increasing availability of P and micronutrients because organic anions could compete with P for complexation by Fe, Al, and Ca [29,55]. However, the nature of root-secreted LMWOAs varied significantly in response to environmental stresses, especially under P nutrient deficiency [21,23,54]. In the present study, concentrations of LMWOAs in root exudates of ryegrass under CPY treatments are summarized in Fig 6d. CPY could significantly increase the acetic, oxalic, citric and malic acids exudation when irrigated with P-nutrient solution, but oxalic acid was the dominant species in root-secreted LMWOAs in NC1 and NC3 treatments (Fig 6d). In our previous work [2], it was found that oxalic acid was capable of inhibiting the precipitation of CPY and enhancing the dissolution of CPY through complexation with surface Pb on CPY. Therefore, the secretion of oxalic acid by ryegrass was probably a specific response to stress deficiency. The present results also suggest that P and Pb uptake and LMWOAs (especially oxalic acid) secretion by ryegrass root might be the driving forces for root-induced dissolution of CPY. Nevertheless, it should be pointed out that many microorganisms could make insoluble soil phosphate bioavailable, for example the dissolution of insoluble metal phosphates by free-living and symbiotic fungi has been reported [56,57]. Therefore, the importance of considering microbial processes when evaluating the long-term stability of CPY in the rhizosphere also needs to be emphasized in future study.

## 5. Conclusions

To achieve the remediation of Pb-contaminated soil, transformation of Pb to extremely stable CPY using P-bearing materials, has gained extensive attention and recognition over the world. Therefore, understanding the stability of CPY has considerable benefits for risk assessment and remediation strategies in contaminated water and soil. In this study, the stability of CPY in ryegrass rhizosphere was evaluated with results showing that the stability of CPY in the rhizosphere may be not as high as previously reported. This reduction in the stability of CPY in the rhizosphere may be due to root exudation of LMWOAs caused by P-deficiency under CPY treatments. Decreases in ryegrass biomass and rhizosphere pH indicated the potential phytotoxicity of CPY. Continuous production of LMWOAs in the rhizosphere would subject CPY to several dissolution reactions that might lead to the possible release of Pb and P into the environment. Root-secreted oxalic acid was firstly introduced by this study to play an important role in root-induced dissolution and reduced the stability of CPY. Remediation technology employing phosphate-induced immobilization of Pb should, therefore, be reconsidered due to the ubiquity of soil LMWOAs, which can release Pb from CPY and increase its bioavailability. Whereas, the effect of other factors, such as dissolved organic matter, on the long-term stability of CPY in the rhizosphere zone, as well as the transport and transformation of CPY in soils, needs further study.

## Supporting Information

S1 File Data for Figs 1–6. (RAR)
